# Exploiting the message from cancer: the diagnostic value of extracellular vesicles for clinical applications

**DOI:** 10.1038/s12276-019-0219-1

**Published:** 2019-03-15

**Authors:** Nobuyoshi Kosaka, Akiko Kogure, Tomofumi Yamamoto, Fumihiko Urabe, Wataru Usuba, Marta Prieto-Vila, Takahiro Ochiya

**Affiliations:** 1 Department of Translational Research for Extracellular Vesicles, Tokyo Medical University, Tokyo, Japan; 2 Division of Molecular and Cellular Medicine, National Cancer Center Research Institute, Tokyo, Japan; 3 Department of Molecular and Cellular Medicine, Institute of Medical Science, Tokyo Medical University, Tokyo, Japan

**Keywords:** Proteomics

## Abstract

Liquid biopsy is indispensable for the resolution of current medical issues, such as the cost of developing new drugs and predicting responses of patients to drugs. In this sense, not only the technology for liquid biopsy but also the target biomolecules for biomarkers need to be identified. Extracellular vesicles (EVs), which contain various proteins, including membrane-bound proteins, and RNAs, including mRNA and long/short noncoding RNAs, have emerged as ideal targets for liquid biopsy. These complex biomolecules are covered by a lipid bilayer, which can protect them from degradation. In this review, we review current topics regarding EVs as cancer biomarkers and introduce technologies used for these recently emerged biomolecules.

## Cancer diagnosis: good things come in small packages

Extracellular vesicles (EVs), small packets of proteins and RNAs secreted by cells and carried in the bloodstream, are promising diagnostic markers for cancer. Developing new markers that can be collected non-invasively can dramatically improve detection and treatment of cancer. EVs secreted by cells, including tumor cells, send messages to distant cells. EVs collected from blood samples can potentially be excellent disease markers. Takahiro Ochiya and Nobuyoshi Kosaka at Tokyo Medical University in Japan and co-workers have reviewed available EV technology. They report that tumors reliably secrete diagnostic EVs, revealing the origin and state of the tumor. Some EVs can even be used to predict whether a cancer might spread to another tissue. With improvement of methods for isolation and standardization, EVs could provide sensitive markers for early diagnosis of many cancers.

## Introduction

To detect cancer in its early stages, clinicians employ a variety of information that can be acquired from the patient’s medical history, imaging data, test results and surgical findings. It is well acknowledged that the early detection of cancer is critical to reduce the physical burdens on cancer patients; early detection reduces patient mortality and increases survival. The ideal diagnostic method accurately and noninvasively detects cancer; thus, body fluid samples may be an ideal source for cancer detection. The following examples have been widely used for this purpose: the carcinoembryonic antigen (CEA) for adenocarcinoma in general, the squamous cell carcinoma antigen for various squamous cell carcinomas, a protein induced by the absence of vitamin K antagonist-II for hepatocellular carcinoma (HCC), the alpha-fetoprotein for yolk sac tumors and HCC, the cancer antigen 125 for ovarian cancer and the prostate-specific antigen (PSA) for prostate cancer (PCa). However, the specificity of these markers is low, and some issues exist regarding false positives and false negatives for diagnosis^1–5^. Thus, these markers are useful for some patients but not for general usage, especially for an early diagnosis. Considering these factors, a novel diagnostic method needs to be developed.

To develop a new diagnostic method, recent research has focused on extracellular vesicles (EVs)^6–10^. A key feature of EVs is that they can be found in various body fluids. Additionally, as we introduce later, EVs are a complex of RNA, proteins and other biomolecules that are covered by a lipid bilayer with transmembrane proteins. In this review, we summarize the current studies regarding EVs as biomarkers for cancer diagnosis based on the biology of EVs in cancer development.

## Definition of EVs

**Fig. 1:**
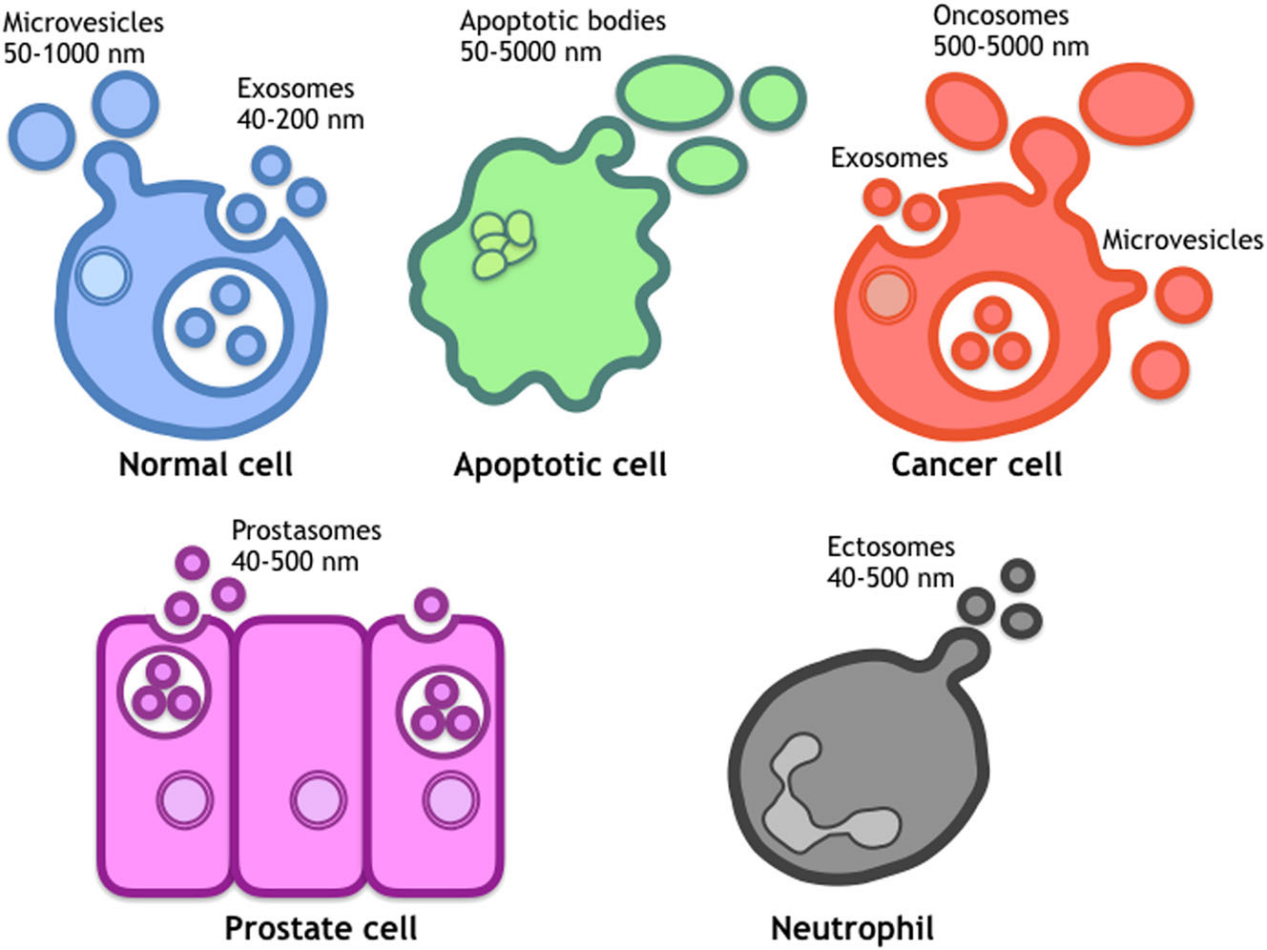
Various types of extracellular vesicles (EVs). Several EVs are released from cells. The sizes cannot properly define each EV, and current knowledge is not sufficient to accurately classify EVs

Because of the complexity of small vesicles derived from biological sources, including exosomes, microvesicles, apoptotic bodies and other types of vesicles, which have been characterized by different research groups and different sources, it is difficult to distinguish small vesicles by the currently available technologies. In addition to this disorganized knowledge about each vesicle type, consensus markers to define these vesicles have not been identified. Thus, the International Society for Extracellular Vesicles (ISEV) has decided to use the term ‘EVs’ as an umbrella term for all types of vesicles that exist in the extracellular space, including exosomes, microvesicles, ectosomes, oncosomes, tolerosomes and prostasomes^11–16^ (Fig. 1). The ISEV has also encouraged researchers to clearly state their method of vesicle collection and how it relates to their terms of use. In fact, EVs are released from various types of cells, including reticulocytes, B and T lymphocytes, dendritic cells, mast cells, platelets, intestinal epithelial cells, astrocytes, and neurons^17–24^. In addition to these normal cells, pathological cells, such as cancer cells, can release EVs. Furthermore, they reflect the features of the origin and state of the tumor. Therefore, it was proposed that EVs could act as novel biomarkers since their discovery in various human body fluids.

## EV-associated proteins

**Fig. 2:**
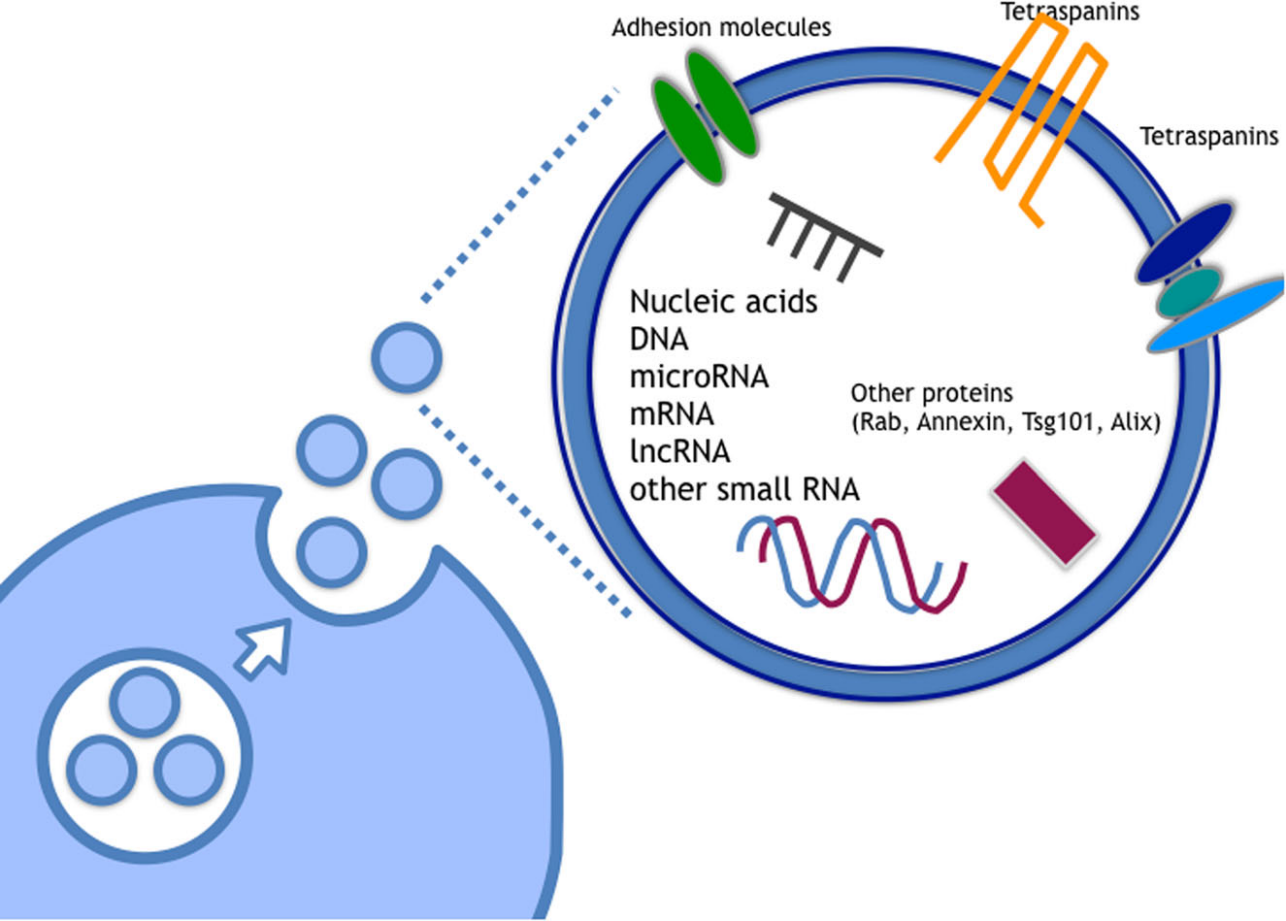
Components of EVs. In EVs, various types of RNAs, such as small noncoding RNA, long noncoding RNA, and coding RNA and DNA are included. Proteins are also contained in EVs and on EV lipid bilayers

Currently, it is well-known that EVs carry multiple types of proteins, such as tetraspanins (CD63, CD9, and CD81), heat shock proteins (HSP70), Rab family proteins, Tsg101 and Alix^25,26^ (Fig. 2). The contents of EVs are influenced by the original cells that secrete EVs; this influence is particularly interesting for biomarkers involved in human disease, especially cancer. As described above, various types of proteins, including membrane and cytosolic proteins, can be found in EVs (Fig. 2), and they are involved in various biological functions^27^. Indeed, it has been shown that the proteins located on the membrane of EVs from cancer cells play roles in cancer progression. For instance, metastatic melanomas release EVs that carry programmed death-ligand 1 (PD-L1) on their surface, which suppresses the function of CD8 T cells and facilitates tumor growth^28^. Furthermore, distinct integrin expression patterns on tumor-derived EVs, distinct from tumor cells, dictate EV adhesion to specific cell types and ECM molecules in particular organs. Additionally, the specific EV integrins in the plasma of cancer patients correlate with and predict likely sites of metastasis^29^.

In recent years, an increasing number of studies have described the use of membrane proteins because they can be captured by antibodies against membrane proteins without destroying the structure of EVs to yield naïve EVs. Furthermore, this method can be used to develop new methods to capture membrane proteins and combine them with other molecules in EVs, leading to a better EV-associated diagnostic target than that of other types of molecules.

Epithelial cell adhesion molecule (EpCAM), which is a transmembrane glycoprotein that is involved in cell-cell adhesion in epithelia, cell signaling, migration, proliferation, and differentiation^30^, may be one of the leading target proteins. Logozzi et al. showed that EV-associated proteins may be used as part of an advanced method to capture target EVs^7^. The authors reported that the membrane protein caveolin-1 on EVs reflects the status of cancer and developed a new technology, ExoTEST, to detect and quantify EVs. ExoTEST is an ELISA-based method that enables the capture and the quantification of EVs in plasma based on the expression of abundant proteins on the EVs (CD63 and Rab5b) and a tumor-associated marker (caveolin-1). Other authors have also shown the detection of EVs from the culture supernatant of human cancer cells and plasma from severe combined immunodeficiency mice engrafted with human melanoma cells. In the mouse model, the level of caveolin-1-positive plasma EVs was significantly increased compared to the control mice. Other authors also validated the ability of ExoTEST to identify EVs expressing CD63 or caveolin-1 in the plasma of melanoma patients. Specifically, levels of these EVs were significantly increased in patients compared with those in healthy donors. Although the sensitivity of this assay was limited (43–68%), this new noninvasive assay exhibited the important indication that the detection and quantification of EVs might require novel noninvasive biomarkers.

Considering the advantages of EVs as biomarkers, various types of body fluids have also been investigated for cancer-derived EVs. Lu et al. found that caveolin-1 and CD59 colocalize with delta-catenin in EVs, and these proteins act to excrete delta-catenin into the extracellular matrix^8^. Delta-catenin, caveolin-1, and CD59 were all detected in the urinary EVs of PCa patients. Furthermore, Bijnsdorp et al. found increased levels of alpha1-integrin and beta1-integrin in urinary EVs of patients with metastatic PCa compared with those in patients with nonmetastatic disease^31^. Various body fluids can be used to test for the presence of cancer, and the use of these body fluids may greatly help improve the quality of life of cancer patients.

**Fig. 3:**
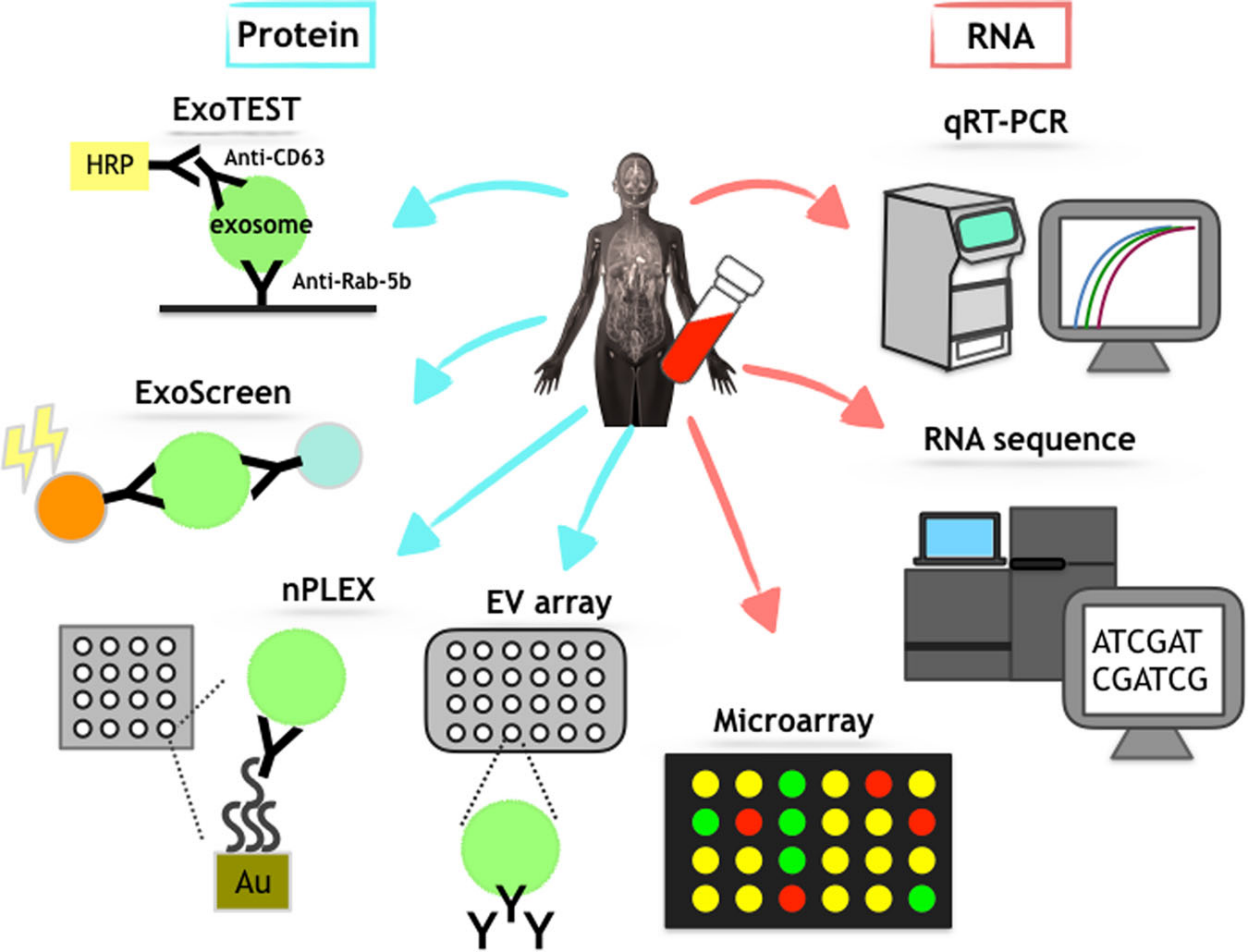
Various types of secreted miRNAs from cells. Several types of circulating miRNAs can be found in human body fluids

As shown above, EV-associated proteins can serve as practical diagnostic molecules. It is noteworthy that EVs contain various types of proteins. Thus, these proteins can be used to develop detection technologies. In fact, new technologies to detect EVs that utilize EV-associated proteins are currently being investigated. The potential of EVs as biomarkers in clinical practice has been widely recognized, but the technology to detect them is poorly developed. Because the isolation and the detection of EVs are typically difficult, currently available technologies to detect EVs in human body fluids for diagnosis are not useful for clinical applications. Thus, technologies that can detect EVs in human body fluids are needed (Fig. 3).

Yoshioka et al^32^. developed the ExoScreen assay, which can exploit circulating EVs in the serum to diagnose early-stage colorectal cancer. This technique can specifically detect circulating EVs in the serum via an amplified luminescent proximity homogeneous assay using photosensitizer beads and two types of antibodies, CD9 and CD147, without the need for any purification steps. It is well-known that CD9 is an EV marker protein, and this assay utilizes CD9 as the target to capture EVs after validating other EV marker proteins, such as CD63. Furthermore, the enrichment of CD147 was found in the EVs of colorectal cancer cell lines and in the serum of colorectal cancer patients; these EVs and serum can be exploited to diagnose early-stage cancer. In summary, this assay detected CD147/CD9 double-positive EVs in the serum of stage I colorectal cancer patients. The level of double-positive EVs was significantly greater in the sera of 194 cancer patients than in the sera of 191 healthy donors. Moreover, the level of CD147 in cancer patient sera decreased after surgery, suggesting that ExoScreen is likely to accurately reflect the profile of EVs derived from the tumor. Additionally, the sensitivity and the specificity of this assay are superior to those of conventional methods based on cancer biomarkers, such as CEA and CA19-9, because these markers were in the normal value range in this cohort. Thus, this system is superior to ELISA and immunoblotting in many respects.

Im et al. developed a high-throughput analysis of proteins for EVs named the nanoplasmonic exosome (nPLEX) assay, which is based on surface plasmon resonance^33^. This method employs a chip called an nPLEX sensor, which has an array of periodic nanoholes patterned on a gold film. The authors analyzed EVs derived from different ovarian cancer cell lines and found EpCAM and CD24 as markers to distinguish among ovarian cancer-derived EVs. Moreover, this method enables the release of captured EVs from the chip to allow their RNA content to be analyzed by quantitative real-time PCR.

An EV array, which is based on the technology of a protein microarray, has been reported^34^. This method enables the detection of EVs from a crude sample in a high-throughput manner. Antibodies against the surface or surface-associated proteins on EVs are printed onto coated glass slides, and then antibodies capture EVs. Next, biotinylated antibodies against CD9, CD63, and CD81 were employed to detect EVs. EVs isolated from the plasma of non-small cell lung carcinoma patients were used to validate the ability of the EV array^35^.

As described above, proteins on the surface of EVs can modulate the technologies described above using common features, including the requirement of a small sample volume and no complicated isolation step. Thus, these technologies enable high-throughput analyses. However, many of these technologies require an antibody that can specifically bind to EVs. Additionally, more studies are needed to validate the reproducibility of these technologies. Thus, identifying specific molecules on EVs for cancer biomarkers is still needed for further investigation.

## EV-associated RNAs

**Fig. 4:**
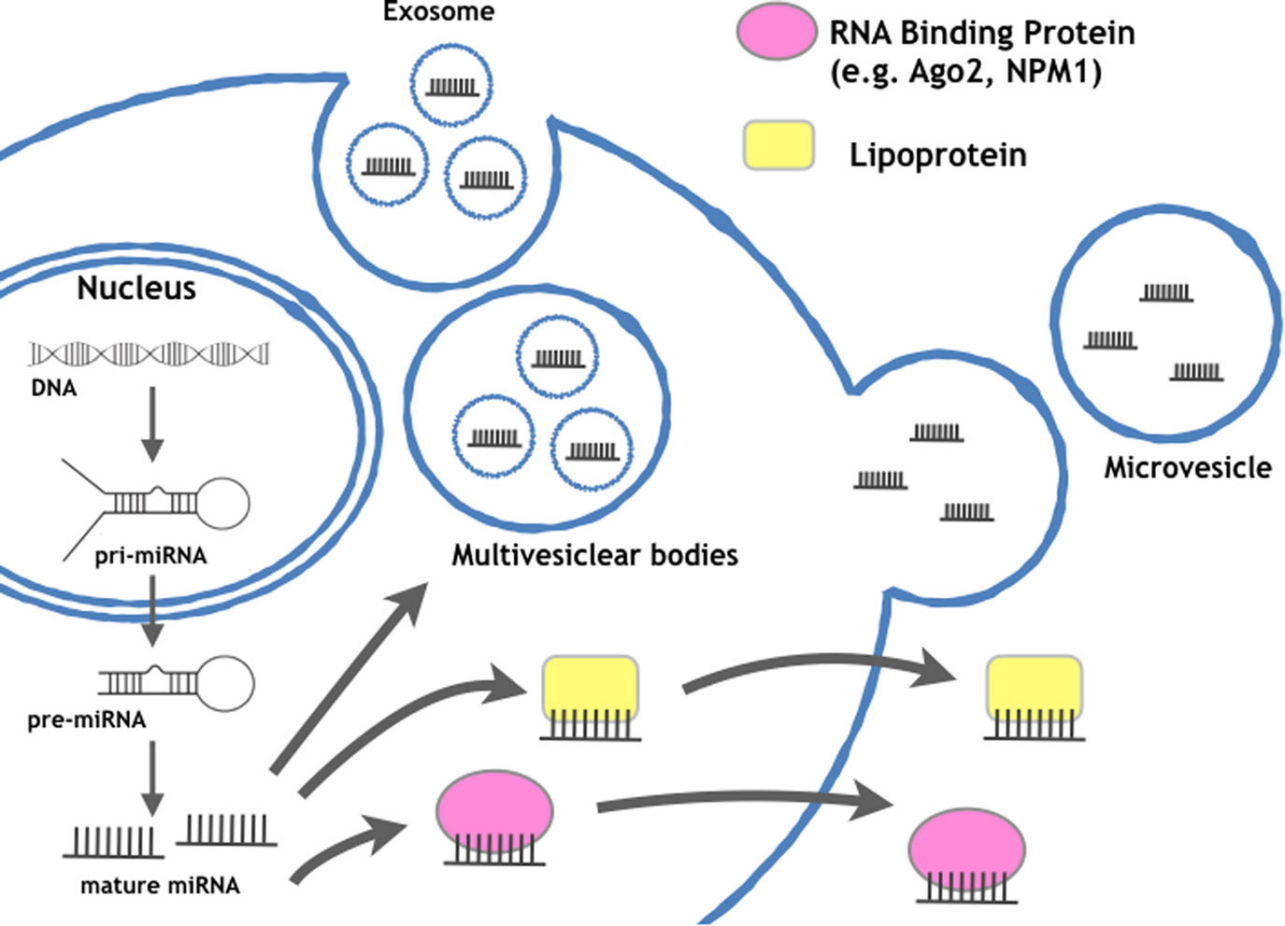
Schematic representation of the current detection technology for biomolecules in EVs. Several methods exist for rapid, high-throughput analyses of EVs. In particular, current technology regarding RNA detection has been developing rapidly

In addition to proteins, RNAs in EVs are also attractive target molecules for biomarkers. EV-associated RNAs, including microRNAs (miRNAs) (Fig. 4), mRNAs, long noncoding RNAs (lncRNAs), and small RNAs other than miRNAs, can be transferred between cells and enact their roles in recipient cells. In 2006, Ratajczak et al. showed that mRNA in EVs released from embryonic stem cells was transferred to hematopoietic progenitor cells and exhibited specific functions^36^. In the following year, Valadi et al. identified miRNAs in EVs derived from human and murine mast cell lines^37^. These pioneering studies shed light on the future of EV research. Subsequently, the function of miRNAs in EVs was proven in target cells, where miRNAs effectively silenced genes^38–40^. Since the discovery of EV-associated mRNAs and miRNAs, many researchers have confirmed the function of RNAs in EVs and have shown novel biology in the various physiological and pathological phenomena mediated by this novel humoral regulator. In addition to these advancements, it has been shown that these EV-associated RNAs may serve as biomarkers because their contents depend on the origin of secreting cells and can be found elsewhere in human body fluids^1,2,41^. Indeed, it has been shown that the systemic injection of brain metastatic cancer cell-derived EVs promotes brain metastasis of breast cancer cell lines and that these EVs are preferentially incorporated into the brain *in vivo*, indicating a novel mechanism of brain metastasis mediated by EVs that trigger the destruction of the blood-brain barrier (BBB)^42^. Additionally, it was revealed that miR-181c in EVs from brain metastatic breast cancer cells promotes the destruction of the BBB through the abnormal localization of actin. Thus, it is possible that miR-181c in EVs from brain-metastasized cancer cells could be found in sera from breast cancer patients who have metastases in the brain due to the leakage of circulating EVs from brain metastatic cancer. Indeed, the miR-181c level in EVs collected from brain metastasis patients’ sera was significantly greater than that in nonbrain metastasis patients. The serum level of miR-181c was significantly greater in the sera from brain metastasis patients (stage IV) than in the sera from nonbrain metastasis patients (stage III and stage IV). Interestingly, in stage IV patients, the miR-181c level was greater in the sera from brain metastasis patients. This result emphasizes that secreting miR-181c is related to the brain metastasis of breast cancer patients. From here, we will introduce several miRNAs that can be detected in body fluids from cancer patients.

The potential of miRNAs in EVs for the diagnosis of esophageal squamous cell cancer (ESCC) has been proposed. Tanaka et al. reported that the level of miR-21 in EVs in the sera from patients with ESCC was elevated compared with that in the sera from patients who harbor benign tumors without systemic inflammation. Furthermore, tumor progression and aggressiveness were positively correlated with the level of this miRNA^43^. Additionally, Takeshita et al. reported that the miR-1246 level in the serum can be used to diagnose ESCC with a sensitivity of 71.3% and a specificity of 73.9%. This significant correlation may be a strong independent risk factor for poor survival with the tumor, lymph node and metastasis stage, as well as the level of miR-1246 in the serum^44^. Although the level of miR-1246 was elevated in the serum from ECSS patients, the upregulation of miR-1246 was not observed in ESCC biopsy samples. These results suggest that the levels of miRNAs in body fluids do not necessarily correlate with the expression from the origin. However, in this study, they only confirmed that the amount of miR-1246 in the EVs among the ECSS cell lines that were evaluated, and it is unclear if the origin of the serum miR-1246 was either the EVs or other circulating miRNAs. In addition to this research, a recent investigation found that miR-1246 in EVs is a promising prostate cancer biomarker with a diagnostic potential that can predict disease aggressiveness^45^. In this study, Bhagirath et al. employed a novel, digital amplification-free quantification method using nCounter technology to profile serum miRNAs in EVs from aggressive prostate cancer cases, benign prostatic hyperplasia cases, and disease-free controls. From this investigation, the tumor suppressor miR-1246 was downregulated not only in serum but also in prostate cancer clinical tissues and cell lines. These two papers indicated that miR-1246 in EVs is a promising cancer biomarker with diagnostic potential that can predict disease aggressiveness.

The most dramatic technology to detect RNA was recently developed, and it is clear that RNA sequencing will be a game changer in future clinical usage. Indeed, the prognostic significance of circulating EV-associated miRNAs in multiple myeloma (MM) from a cohort of 156 patients with newly diagnosed MM was examined by small RNA sequencing analysis^46^. The miRNAs let-7b and miR-18a in EVs from serum samples were significantly associated with both progression-free survival (PFS) and overall survival (OS) in their univariate analysis. Thus, the authors concluded that the use of circulating exosomal miRNAs could improve the identification of patients with newly diagnosed MM with poor outcomes. Furthermore, it was shown that EV-associated miRNAs reflect metabolic disease in classical Hodgkin’s lymphoma (cHL) patients. In that study, the authors isolated EVs with standardized size-exclusion chromatography from the plasma of cHL patients and healthy subjects and then performed a comprehensive small RNA sequencing analysis^47^. The authors found that purified EV fractions of untreated cHL patients and tumor EVs had enriched levels of miR24-3p, miR127-3p, miR21-5p, miR155-5p, and let7a-5p compared with EV fractions from healthy subjects and disease controls. Serial monitoring of the EV miRNA levels in patients before treatment, directly after treatment, and during long-term follow-up revealed robust, stable decreases in miRNA levels matching a complete metabolic response, as observed with FDG-PET. Importantly, EV miRNA levels rose again in relapse patients. Thus, cHL-related miRNA levels in circulating EVs reflect the presence of vital tumor tissue and are suitable for therapeutic response and relapse monitoring in individual cHL patients.

It is noteworthy that mRNAs in EVs could not only be functional molecules that become proteins in recipient cells but also diagnostic tools for cancer detection. Recently, Yokoi et al. revealed novel mechanisms for metastasis mediated by EVs in ovarian cancer^48^. In their study, they found that MMP1 mRNA in EVs from ovarian cancer-induced apoptosis in mesothelial cells, leading to the peritoneal metastasis of ovarian cancer cells. Importantly, they identified the EVs carrying MMP1 mRNA in patient-derived ascites and proposed their use as an indicator of peritoneal dissemination risk, even in the early stages.

The contribution of other noncoding RNAs, such as long noncoding RNA (lncRNA), in EVs from cancer cells is becoming increasingly recognized. Additionally, accumulating evidence suggests that lncRNAs in EVs can also be biomarkers for cancer. Kogure et al. demonstrated that HCC cell-derived EVs contain ucRNA (ultraconserved RNA), which is a group of lncRNAs transcribed from ultraconserved elements whose genomic sequences are greater than 200 bases and are 100% conserved across human, mouse, and rat genomes;^49^ they showed that these EVs can be taken up by other HCC cells, resulting in the intercellular transfer of ucRNA with subsequent modulation in cellular function. Thereafter, several reports demonstrated their biological importance not only as intermediators of cell-cell communication but also as biomarkers for disease detection. Indeed, Wang et al. showed that the expression of miR-21 and HOTAIR in EVs was significantly higher in patients with laryngeal squamous cell carcinoma (LSCC) than in patients with vocal cord polyps. Significant differences were found in serum miR-21 and HOTAIR expression in EVs between the advanced T classifications (T3/T4) or clinical stages (III/IV) and the early stages of LSCC. The patients with lymph node metastasis had higher serum miR-21 and HOTAIR expression in EVs than those without lymph node metastasis^50^. Additionally, Zhao et al. examined the serum long noncoding RNA HOTTIP in EVs from 126 gastric cancer (GC) patients and 120 healthy controls and found that the expression levels of HOTTIP in EVs were typically upregulated in GC compared with those in normal controls^51^. Furthermore, expression levels of HOTTIP were significantly correlated with the invasion depth and TNM stage, suggesting that HOTTIP in EVs is a potential biomarker for GC in diagnosis and prognosis.

Other small noncoding RNAs besides miRNAs were also examined for their usefulness as cancer biomarkers. Indeed, RNA sequencing and proteome analyses of chronic lymphocytic leukemia (CLL)-derived EVs were performed, and noncoding Y RNA hY4 in EVs from the plasma of CLL patients was abundant compared with that from healthy donor samples^52^. Interestingly, the CLL-derived exosomal Y RNA, hY4, promotes monocytes in CLL patients to adopt an immunosuppressive phenotype, including promoting the expression of PD-L1, and showed that hY4 promotes exosome-dependent skewing of monocytes in a TLR7-dependent manner. Again, it is noteworthy to indicate that RNAs in EVs are stable in human body fluids because they are resistant to RNases, which are abundant in human body fluids. Therefore, this resistance indicates several advantages, such as reproducibility, storage, and sensibility, to using EV-associated RNAs as cancer biomarkers. Additionally, several technologies, including qPCR and microarray RNA sequencing, can detect nucleic acids. Considering the availability of these technologies, nucleic acids in EVs are an ideal resource for cancer diagnosis. Furthermore, noting the advantage of EVs as a complex of biomolecules, cancer-specific miRNAs can be detected by utilizing other cancer-specific molecules in membrane proteins, resulting in the detection of highly specific cancer biomarkers. Considering these advantages, further studies of various RNAs in EVs are essential for clinical applications.

## Future perspectives

In this manuscript, we have shown the potential for EVs in the development of cancer biomarkers. As stated in the introduction of this manuscript, EVs are a complex of multiple biomolecules, including small, long coding and noncoding RNAs and proteins, that provide useful information for clinicians. The enrichment of cancer-specific EVs from blood, as well as the rapid, on-chip analysis of their RNA contents, has been developed and named ‘immuno-magnetic EV RNA analysis’^53^. Several biomolecules, such as DNA, glycans, lipids, and metabolites, have not been discussed in this manuscript. Thus, it is essential to continue the investigation to clarify the importance of the diagnosis of EV contents, which could provide powerful tools for cancer diagnosis. Additionally, it is crucial to develop new platforms to capture EVs.

It is difficult to determine the most suitable targets for cancer biomarkers. Indeed, it has been shown that glypican-1 is a biomarker to detect pancreatic cancer^54^. However, a recent report indicated that the miRNA signature in EVs is superior to GPC1 in EVs or plasma CA 19-9 levels in establishing a diagnosis of pancreatic ductal adenocarcinoma and differentiating between pancreatic ductal adenocarcinoma and chronic pancreatitis^55^. It is too early to determine which result is correct because several issues need to be resolved concerning EV research; however, for the realization of EV-based cancer biomarkers, further research focusing on these issues is necessary.

In this manuscript, we introduced several technologies, such as ExoScreen, nPLEX and EV array, to capture EVs. Although research on EVs as cancer biomarkers has been actively performed, basic aspects of EV research are not fully clarified. First, the pathophysiological roles of EVs remain unclear. For instance, basic knowledge regarding the sorting mechanism for the content of EVs, the secretion mechanism, and the uptake mechanism has not yet been revealed. This knowledge is crucial for understanding the importance of EVs in physiological status by generating a knock-out mouse. Another issue is the confusion regarding the method for EV isolation. Thus far, several procedures for isolating EVs, such as ultracentrifugation-, column- or polymer-based methods, have been utilized. Thus, it is essential to understand the pros and cons of each method. Ueda et al. developed anti-CD9 antibody-coupled, highly porous monolithic silica microchips. In this column, the authors isolated EVs from 46 serum samples from lung cancer patients. Subsequently, they performed mass spectrum chromatography and then identified CD91 as a lung adenocarcinoma-related antigen^56^. From this point of view, a novel method for isolating EVs without a complicated isolation method is essential for the further development of EV research to develop biomarkers for cancers. Finally, a reference must be carefully assessed for the normalization of all methods. Some reports have used miR-16 as a reference, and this miRNA may serve as a standard^57,58^. A reference RNA or protein might contribute to the standardization of EV-based biomarkers used worldwide.

Research on EVs as cancer biomarkers is increasing. Although there are many issues to be solved, EVs might be the solution to current medical issues. Because features of EVs have many merits in liquid biopsy, further investigations to identify more accurate and more specific markers, including RNAs and proteins, are essential.

As mentioned above, EV-associated RNAs or proteins that can serve as detection targets will continue to be identified, and the accumulation of these data will lead to the development of a consensus EV marker for each cancer that is highly sensitive and specific. We believe that several clinical trials to employ EVs in the diagnosis of cancer will begin over the next five years. The cost of medicine is the largest issue worldwide, and a recent report has shown that the novel technology for EV-based diagnosis seems to be inexpensive because the technology employed is simple. Additionally, a well-established diagnosis for cancer by targeting EVs will be employed in the near future. Furthermore, there are several challenges that try to use or target EVs in cancer^59–61^. Although the roles of EVs in pathophysiological conditions have not yet been clarified, an EV-associated diagnosis will soon be employed.
